# *I’ve lost the person I used to be—*Experiences of the consequences of fatigue following myocardial infarction

**DOI:** 10.3402/qhw.v8i0.20836

**Published:** 2013-06-14

**Authors:** Ulla Fredriksson-Larsson, Pia Alsen, Eva Brink

**Affiliations:** 1 Department of Nursing, Health and Culture, University West, Trollhättan; 2 Institute of Health and Care Sciences, University of Gothenburg, Gothenburg, Sweden; 3 University of Gothenburg Centre for Person-Centred Care (GPCC), Sahlgrenska Academy, University of Gothenburg, Gothenburg, Sweden

**Keywords:** Fatigue, grounded theory, myocardial infarction, symptom experience, person-centered care, sense of coherence

## Abstract

Fatigue has been found to be the most frequent and bothersome symptom after myocardial infarction (MI), influencing health-related quality of life negatively. Moreover, fatigue after MI has been described as incomprehensible due to its unpredictable occurrence and lack of relationship to physical effort. The aim of this study is therefore to explore persons’ experiences of consequences of fatigue and their strategies for dealing with it 2 months after MI. In total, 18 informants, aged 42–75 years, participated in the study. Interviews were conducted and analysed using constructivist grounded theory methodology. Grounded in the data, the main consequence of fatigue, as illustrated in the core category, was: *I’ve lost the person I used to be*. It indicates a sense of reduced ability to manage daily life due to experiences of fatigue. The core category was developed from the four categories: *involuntary thoughts, certainties replaced with question marks, driving with the handbrake on* and *just being is enough*. Furthermore, attempts to relieve fatigue were limited. These findings indicate that patients with symptoms of fatigue should be supported in developing relief strategies, for example, rest and sleep hygiene as well as physical activity. In conclusion, the results show that fatigue can be understood in light of the concepts “comprehensibility” and “manageability.” They also indicate that, working from a person-centered perspective, health-care professionals can support patients experiencing post-MI fatigue by giving them opportunities to straighten out the question marks and by inviting them to discuss involuntary thoughts and feelings of being restricted in their daily life functioning.

Illness is a subjective experience, often expressed in terms of symptoms. It is worth keeping in mind, however, that there is not always congruence between symptom experiences and specific diagnoses (Ekman, Cleland, Andersson, & Swedberg, 2005). The symptom fatigue is poorly defined, despite the fact that persons commonly regard fatigue as a serious and disabling experience. In contrast, health-care professionals sometimes see fatigue as a diagnostically non-specific state (Sharpe & Wilks, 2002). Consequently, although fatigue has a major impact on daily life, it often goes unnoticed and underdiagnosed (Payne, Wiffen, & Martin, 2012).

Many persons in primary care settings and with a range of diagnoses have reported fatigue as their most common symptom (Nijrolder, Van der Windt, De Vries, & Van der Horst, 2009). Fatigue has been explored in several studies including persons with heart failure. These studies have shown that fatigue is a distressing symptom that negatively affects activities in daily life (e.g., Jones, McDermott, Nowels, Matlock, & Bekelman, 2012; Norberg, Boman, & Löfgren, 2010). Research on fatigue after myocardial infarction (MI) is more limited, although fatigue has been reported to be the most frequent (Kristofferzon, Löfmark, & Carlsson, 2007) and bothersome symptom (Brink, Karlsson, & Hallberg, 2002), with negative consequences for engagement in physical activity (Galdas, Oliffe, Kang, & Kelly, 2012). Research has also shown that fatigue influences health-related quality of life negatively (Brink, Alsén, Herlitz, Kjellgren, & Cliffordson, 2012). Persons in the early rehabilitation phase after MI described an inability to perform basic activities of daily living, for example, shopping or gardening, due to fatigue and other health problems. In addition, symptoms of fatigue had an impact on daily life activities owing to the need for daily rest (Roebuck, Furze, & Thompson, 2001).

According to Ream and Richardson (1996, p. 527), fatigue is defined as a “subjective, unpleasant symptom that incorporates body feelings ranging from tiredness to exhaustion, creating an unrelenting overall condition that interferes with persons’ ability to function to their normal capacity” (Ream & Richardson, 1996). Tiredness can be an indication of a progression to fatigue, but should not be considered synonymous with fatigue. Persons’ behavioral patterns in relation to tiredness, for example, sleep quality, stamina, physical condition and emotional reactivity, could serve as very early signs of impending fatigue (Olson, 2007).

Fatigue is a core symptom of major depression according to DSM-IV (American Psychiatric Association, 2000). When depression is the main cause of fatigue, anti-depressive therapy may be required. However, in a sample of persons treated for MI 4 months earlier, it was shown that several persons experienced fatigue without depression and that half of the group reported fatigue (20% with possible or probable depression, 30% fatigue without co-existing depression; Alsén, Brink, Brändström, Karlson, & Persson, 2010). Therefore, persistent fatigue should be emphasized in the rehabilitation of persons treated for coronary heart diseases (Appels, 2004). It would seem beneficial to discuss fatigue relief strategies with a patient who is experiencing fatigue after an MI. There is a great deal that these persons can do themselves. However, to be trustworthy and professional, health-care staff need to know more about post-MI fatigue and its consequences for daily life functioning.

After MI, fatigue was described by persons as incomprehensible due to its unpredictable occurrence, lack of relationship to physical effort and different character as compared to tiredness. Fatigue was described as causing feelings of being restricted and defeated, and the sense of defeat was caused by persons’ sense of having no strategies for fatigue relief (Alsén, Brink, & Persson, 2008). Therefore, the aim of this study is to explore persons’ experiences of the consequences of fatigue and strategies for dealing with fatigue 2 months after MI.

## Methods

This study is based on data from interviews conducted with persons who recently experienced an MI. The interviews were used to gather rich, solid data on which to base a meaningful analysis. In the interviews, the informants revealed their views, feelings, intensions and actions as well as the context and structure of their lives. The qualitative method used was based on constructivist grounded theory methodology (Charmaz, 2006). The method emphasizes examining processes rather than studying the meaning of the phenomenon. In this study, the persons’ experiences of the consequences of fatigue and strategies for dealing with it were analysed, making the study of action central, and for creating abstract interpretive understandings of the data. People construct their realities based on their shared experiences. Interpretation of the studied phenomenon is itself a construction, as is the resulting theory (Charmaz, 2006). The constant comparative method is essential in grounded theory analysis. The method entails that every aspect of the data, for example, emerging codes, categories and properties, be constantly compared with all other aspects of the data, allowing the exploration of variations and similarities (Hallberg, 2006).

### Participants

This study, which is part of a larger research project, comprised a total of 18 informants (five women). The informants were strategically selected and invited from a group of 165 consecutive patients who had been treated for MI 2 months earlier at a rural regional hospital in western Sweden during the period March 2011–March 2012. The informants agreed (verbally and through written informed consent, including questionnaires, diaries and interviews) to participate in a study of fatigue after MI. Prospective informants were excluded if they were older than 75 years, had a communication disability such as difficulty understanding and speaking Swedish, and/or had disabilities resulting from, for example, dementia or stroke. Persons with serious illnesses such as advanced cancer were also excluded. Two months after MI, the informants answered a written questionnaire, the Multidimensional Fatigue Inventory (MFI-20), which measures fatigue in five dimensions (general fatigue, physical fatigue, reduced activity, reduced motivation and mental fatigue (Smets, Garssen, Bonke & De Haes, 1995)). The 18 informants in this study were strategically selected from those with values above 13/20 on one or more dimensions of the MFI scale. Descriptive data on the 18 informants are presented in Table I.

**Table I:** Characteristics of the 18 informants.

Informants	Sex	Age	Professional	Marital status
1	Male	46	Managing director	Single
2	Male	42	Truck driver	Cohabitant
3	Male	65	Former lecture	Married
4	Male	69	Former managing director	Married
5	Female	75	Former secretary	Single
6	Female	66	Former maid	Married
7	Female	66	Former nurse	Cohabitant
8	Male	66	Truck driver	Married
9	Male	53	Managing director	Cohabitant
10	Male	52	Unemployed	Cohabitant
11	Male	65	Pensioners	Married
12	Female	67	Housewife	Married
13	Female	57	Nurse	Married
14	Male	60	Attendant	Married
15	Male	56	Caretaker	Cohabitant
16	Male	57	Workers	Married
17	Male	61	Administrator	Married
18	Male	60	Police	Married

### Procedure/interviews

The 18 informants were contacted through telephone and asked to participate in an interview about fatigue after MI. Where and when the interview would take place was agreed on at this point. Four interviews took place in the informant’s home, six in a private room in a primary care or hospital facility, four in a private room at the university, two in private rooms at the informant′s workplace and two in separate rooms in a public restaurant. The interviews, which lasted 45–120 min, were audiotaped and transcribed verbatim by the first author (UF-L). The interviews focused on the informant’s own experiences of the consequences of and strategies for dealing with fatigue. To introduce the topic, an initial open-ended question was posed: “Can you tell me what happened when you had your heart attack?” This question reflects an emphasis on learning about each participant’s views, experiences, events and actions. Based on the response to this question, follow-up questions were asked. To encourage the informants to talk and add details, the interviewer said: “Please, tell me more about this”. A few broad questions were asked: “Tell me about a day when you feel tired, what do you think about, and what do you do?”, “What are the consequences of fatigue and how do you manage them?”, “How do you perceive your recovery process?”

### Data analysis

Analysis of the interviews took place as soon as an interview had been conducted and subsequently transcribed. Each interview was read through to get a sense of the whole. In line with grounded theory methodology, the interviews were analysed meanwhile working with data collection. In line with constructivist grounded theory, the analysis was carried out through initial coding and focused coding (Charmaz, 2006). During the initial coding, which was done line-by-line close to the data, segments of the data that included experiences and expressions of actions were defined. By using the informant’s own words, we ensured that these codes were grounded in the data. Next, the initial codes were labeled, and comparisons between the data and the emerging categories were made. Data collection progressed simultaneously with this initial analysis and the coding process. The emerging pattern was examined, and categories were refined.

In the focused coding, the most significant codes were selected, and through identification, verification and specification of relationships between the categories, a core category was created. During the entire process, theoretical reflections, questions and ideas based on the data were written down in memos (Charmaz, 2006). The first author conducted the analysis in collaboration with the second and third authors.

### Ethical considerations

The study was approved by the Regional Ethical Review Board in Gothenburg (720-10). The informants were informed both verbally and in writing about the study aim and procedures. Written and oral informed consent were obtained from those who wished to participate in the study.

## Results

The identified core category, *I’ve lost the person I used to be*, integrates the categories grounded in the data, providing a foundation for understanding the consequences of post-MI fatigue. The categories were labeled: *involuntary thoughts, certainties replaced with question marks, driving with the handbrake on* and *just being is enough*. The core category implies a sense of diminished ability to manage daily life due to experiences of fatigue. The new feeling—of having lost the person I used to be—is characterized as a personal challenge, uncertainty, reduced motivation and difficulties controlling one’s thoughts. “Fatigue has changed me a lot, I’ve never been like this. That’s why I hardly recognize myself now. (p. 12)”

**Figure 1:**
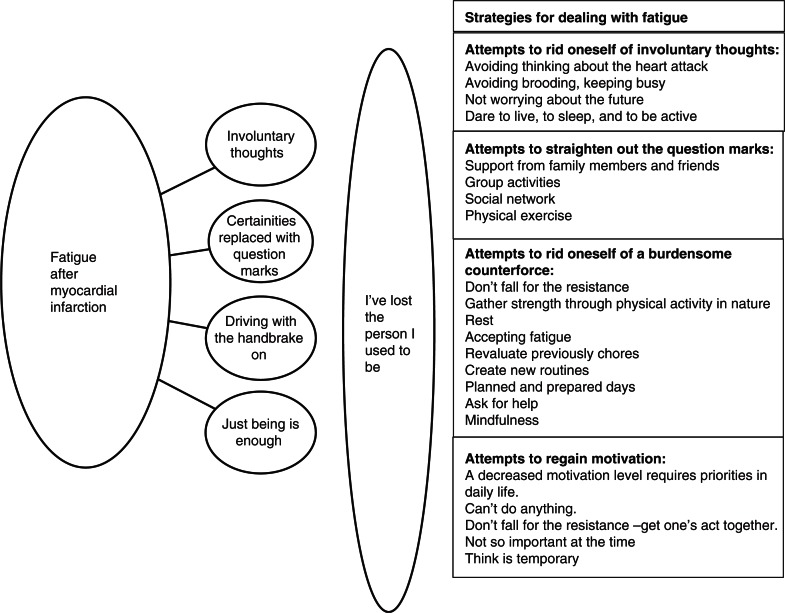
Experiences, consequences, and strategies of fatigue following myocardial infarction.

Also, the results showed fumbling attempts to deal with the effects of fatigue in daily life denoted as attempts to: rid oneself of involuntary thoughts, straighten out the question marks, rid oneself of a burdensome counterforce and regain motivation. Below, the content of the core category is presented more completely, starting with the first category followed by the attempts to deal with fatigue, the next category, and so on; see Figure 1 also.

### Involuntary thoughts

The category *involuntary thoughts* describes experiences of loss of control over one’s own thoughts. The uncontrolled thoughts appeared to be due to fatigue during early rehabilitation from MI and were described as intermittent and occurring in connection with experiences of strong fatigue, but also during certain activities, for example, computer work or reading a newspaper. Thoughts that were described as recurring periodically and as difficult to suppress included unpleasant reflections on life.

I have horrible thoughts it’s like I’ve aged 15 years and I feel so old like life has just passed me by, like everything is over and I can’t do anything about it. (p. 3)

Involuntary thoughts occupy one’s mind and give a feeling of discomfort, but also frustration about not being able to handle them.

It’s all this brooding that’s dangerous, and then the uneasy feelings come along and then I lie down and sleep a while. I’m usually a bit tired then. (p. 12)

#### Attempts to rid oneself of involuntary thoughts

Actions taken to avoid brooding included keeping busy, avoiding thinking about the heart attack incident and not worrying about the future. The intentions expressed were to dare to live, to dare to sleep and to dare to be active. “What’s important now is to get going again, to not think about it … I think, to start brooding over the fact that you’ve had a heart attack and get scared, I don’t think that’s good. Dare to sleep, dare to exert yourself. I’ve tested it and it works, exerting myself, lifting and carrying. (p. 12) 

### Certainties replaced with question marks

The category *certainties replaced with question marks* describes changes that reduced security and instead increased uncertainty in many different ways. Previously obvious positive thoughts about the future have been replaced with questions like: Why am I so tired? How long will I experience fatigue? And how will the fatigue affect my future? Informants experienced uncertainty about returning to daily life because, owing to the fatigue, both their mental and physical strength were unknowns. This made it difficult to plan for the future.

I sat down and thought about it all. What my current situation is like and what it might be like in the future. Because what do I know it might change entirely after a year. I might be like I was before. That’s the idea, right? (p. 1)

The informants described uncertainty about returning to daily life activities, and still had unanswered questions: How is my experience of fatigue associated with myocardial infarction? What level of physical capacity do I have? What level of physical activity is advisable? What the balance was between a healthy return to daily life activities and behaving dangerously was not clear to them.

I might be lying in bed before I get up and think now I’d like to go out for a bike ride, but when I get to the garage and see my bike I don’t know if I can manage it. (p. 4)

Vagueness about what constitutes recommended healthy behavior led to avoidant behavior, with a risk for inactivity and deteriorated health. “I don’t dare do too much at home; my family tells me I shouldn’t exert myself. (p. 15)

Also, informants felt abandoned, because there was no help available for answering questions about their perceived fatigue. “No, there is no help available. I’m thinking that it must just get better by itself, of course, that things will get better. (p. 7)”

#### Attempts to straighten out the question marks

Actions taken to straighten out the question marks were limited. Informants who had participated in group activities, for example, “heart school,” and physical exercise talked about the positive effects of group exercise on fatigue. They also talked about support from family members and friends. However, they expressed a feeling of uncertainty about how to manage daily life due to problems of fatigue. The informants felt that fatigue after MI was unrecognized by health-care professionals, and they suffered due to their lack of knowledge about fatigue and fatigue relief strategies.

### Driving with the handbrake on

Characteristic of the category *driving with the handbrake on* is a current will to be active, but an unexpected counterforce in the form of fatigue that prevents them. This is a new, uncontrollable and bothersome experience of decreased capacity to initiate activity. Despite perceived enthusiasm and motivation, the informants reported that this counterforce created frustration and amazement in themselves and family members. They said they were unable to act and therefore negotiated with themselves to put off tasks for another day.

The worst thing now is that I feel so good and have so many fun things I want to do. I get so frustrated when I don’t get going, there’s so much I want to do that I don’t do anything, that’s more how it is. It’s not the fatigue but initial difficulties in doing things. (p. 11)

Another informant said: but that’s what’s so strange I’m sitting here now and I’m fatigued and don’t want to get going …. it’s like my starting run is long. (p. 1)

The new unexpected feeling of fatigue as a counterforce was difficult to understand and therefore triggered negative feelings of sadness and irritation. The loss of available energy to act was confusing and sometimes interpreted by individuals as a possible symptom of depression.

So I think I’m tired. I have a hard time getting things started. I thought I was depressed, but maybe it was extreme tiredness. I don’t think it was depression so much as just being tired, and I still am. Getting started is difficult. Otherwise I’m very active, but I know that when I get home and sit down, then I can only sit there. I don’t get up again. (p. 15)

Another informant said: It was no problem other than that I was really tired. I didn’t understand. They probably mentioned it but I guess it went in one ear and out the other. Because as soon as I did something, as soon as I lifted something, I was wiped out. I had no strength left in my arms or anything. (p. 17)

#### Attempts to rid oneself of a burdensome counterforce

Strategies described as attempts to rid oneself of feelings of a burdensome counterforce were to struggle to maintain daily tasks and physical activities at the same level as before the heart attack.

I have to exercise to build myself up and counteract the fatigue, because the more I do the easier it is. If I lie down or sit down and feel sorry for myself then I sink quickly you know, my physical condition and energy and all that … and I can’t do that instead I have to make the illness easier by keeping going like normal although there’s a lot of resistance. (p. 5)

New routines were established and each day had to be planned and organized carefully to ensure that one had enough energy, all day long.

Sort of difficult to accept that I need to use strategies all the time, that I have to figure things out ahead of time, can I manage this or should I put it off. I don’t want to get in over my head. It might also have something to do with age. (p. 5)

Some informants had participated in a mindfulness course and had learned to focus on what is essential in life. They therefore attempted when needed to reduce previous demands, reevaluate previous daily work and accept the rest. “It will be Christmas regardless of whether I clean the windows. (p. 12)” It was sometimes necessary to ask for help, even in situations they previously managed by themselves. “I’ve always had trouble asking for help, but recently I’ve actually learned how to. Otherwise it’s: I can do it myself. (p. 15)”

### Just being is enough

The category *just being is enough* describes a new experience of fatigue including decreased motivation and energy only sufficient for “just being”; that is enough. Everyday tasks that were previously pleasant were no longer performed with pleasure. Their physical strength might exist, but commitment and motivation had diminished. Compared to the period before the MI, the respondents described having a more passive approach without any drive and power.

It’s just that I take things more slowly and have less enthusiasm than I did before; I take things as they are. But unfortunately I don’t have goals like I used to, and many people think this is dangerous. It’s really horrible, but like I said I hope I’ll be more like I was with time. (p. 1)

Some of the informants blamed themselves for being lazier now than they had been prior to the MI. “Well I guess I’m simply too tired so that I don’t want to or can’t be bothered, I’m lazy. (p. 14)”

Informants also described diminished social activity due to fatigue. Their motivation to interact with others, such as in family celebrations and activities with grandchildren, was reduced.

It’s not fun anymore what was fun once is not fun now. I’ve changed my mind a lot. (p. 3)

I’m slower, more tired. I actually don’t want to live, but I don’t want to hurt myself either. I want to live like normal, that’s all. Before the MI, I could do three things at the same time, now I have to force myself to take a shower and go out … even if I have to crawl. (p. 6)

Some informants described physical improvement after MI treatment. But despite this, problems with fatigue had a significant impact on their motivation.

I think I notice a difference in that before I had my heart attack I was more tired and sluggish in general but now suddenly you’re physically healthy … and then the other thing is more noticeable because if I’d continued being sick then I wouldn’t have noticed the difference because then my body was in bad shape but the fatigue is still around, like a veil. I think that’s the problem, well problem, I think that’s what’s happened. But now I think it’s hard to find any motivation. (p. 11)

The informants described feelings of sadness and suffering after the experience of having survived a heart attack.

#### Attempts to regain motivation

According to the informants, a decreased motivation level requires making new priorities in daily life.

My lack of motivation means I have to focus on things I think are important, what I want to do. There are some things I feel I have to do. (p. 11)

One strategy the informants reported using was to “not give in to the resistance” but instead to pick oneself up by one’s bootstraps and make oneself do things. Others did not feel that fatigue played such a vital role in daily life, because it is temporary and because what one was planning to do was not so important at the moment.

I pick myself up by the bootstraps and do it anyway but it doesn’t feel good … here inside, it’s no good that I’m like this. (p. 12)

Life has changed because the fatigue is considerable and because bouts of fatigue are unpredictable and difficult to cope with.

I can’t do anything about the fatigue. It comes when it comes. I hope I’m doing what I can. I try to get to bed in time anyway. (p. 10)

## Discussion

The main finding of this study, illustrated by the core category *I’ve lost the person I used to be*, showed that, 2 months post-MI, fatigue was still negatively affecting informants’ ability to manage daily life. The core category emerged from four categories: *involuntary thoughts*, which describes loss of control over one’s own thoughts, *certainties replaced with question marks*, which refers to uncertainty about returning to daily life, *driving with the handbrake on*, which describes an impaired ability to act, and *just being is enough*, which refers to a reduced amount of energy.

Moreover, the results showed fumbling attempts to deal with the effects of fatigue in daily life, denoted as attempts to *rid oneself of involuntary thoughts, straighten out the question marks, rid oneself of a burdensome counterforce and regain motivation*.

The core category in this study showed that fatigue after MI influenced the informants’ feelings about their own normal existence. Informants felt different from how they had been as persons prior to the MI. Based on similar results, Tod (2008) described the meaning of recovery from MI as *watchful insecurity*, relating to the fact that persons felt different compared to how they had felt before the heart attack. Our interpretation is that fatigue contributes to the feeling of having *lost the person I used to be*. This was also supported in an earlier study showing that fatigue restricted bodily, cognitive and affective functioning (Alsén Brink, & Persson, 2008). Further research is needed to investigate whether other factors are involved in this process.

The results showed that informants experienced uncertainty, and previous thoughts about the future were replaced by many unanswered questions. Hospitalization after MI is often short (Stenestrand, Lindbäck, & Wallentin, 2006), and time for patients to ask questions is therefore limited. However, in the early rehabilitation phase, patients are commonly invited to revisit the doctor and sometimes to the rehabilitation nurse, and these visits provide opportunities for questions. One might wonder why so many questions remain unanswered after 2 months. One explanation could be that new questions arise constantly as patients return to daily life activities, and there is no support from health-care professionals during this period of rehabilitation. Fatigue after MI has not received sufficient attention, and the current lack of knowledge about MI fatigue limits health-care professionals’ ability to provide correct information (Appels, 2004). Another possible insight into the issue of uniformed patients was provided in a study of post-stroke fatigue, which discussed the possibility that patients are uninformed because, owing to mental fatigue, they are unable to remember the information they were in fact given (Zedlitz, Van Eijk, Kessels, Geurts, & Fasotti, 2012). Also, women who had received information in hospital about their disease and lifestyle modification found it difficult to understand how this information could benefit their health process. They were not prepared to handle the information because they were still caught up in trying to understand what had happened to them (Johansson & Ekebergh, 2006).

This study showed that the new feeling of having “lost the person I use to be” could have a negative impact on patients’ rehabilitation process. This is in line with research on women’s experiences following MI, which revealed the importance of dealing with the new situation (Johansson & Ekebergh, 2006). The women described a desire for “a normal life” and well-being, but this was complicated by their sense of being lost. They did not know where they could find support—a result also revealed in this study. Fatigue after MI has been described as incomprehensible (Alsén et al., 2008). This study supports this description, and the consequences of fatigue described in the core category indicate that something has to be done to support patients with these experiences. The categories described could be considered in light of the concept of sense of coherence, which reflects a person’s ability to assess and understand the situation, to find a meaning in moving toward health promotion and to then actually do so. According to Antonovsky (1993), a sense of coherence includes the factors “comprehensibility,” “meaningfulness,” and “manageability.” In our results, the informants’ reports of *certainties* having been *replaced with question marks* could be interpreted as lowered comprehensibility. Comprehensibility refers to the extent to which one perceives stimuli as being structured in a predictable and explicable manner. Because the informants experienced vagueness, for example, as to what was considered healthy behavior, their resources were limited—that is, manageability was negatively affected. Manageability refers to the extent to which one perceives that the resources available are sufficient to meet one’s life demands.

Earlier research on coping after MI has not explicitly emphasized fatigue, but there are similarities in descriptions of the recovery process. This study explored fumbling attempts to handle fatigue. This is in line with a recent study of coping orientations after myocardial, showing that coping is threatened if the person denies the seriousness of the situation (Salminen-Tuomaala, Åstedt-Kurki, Rekiaro, & Paavilainen, 2012a). Also, the phenomenon Seeking lost control described by Salminen-Tuomaala, Åstedt-Kurki, Rekiaro, and Paavilainen (2012b) could correspond to the category *Attempts to straighten out the question marks* in this study, meaning an attempt to make sense of the situation or increase comprehensibility.

These findings imply that when a person cannot master daily life demands due to fatigue, health-care professionals should attempt to increase comprehensibility by giving opportunities for the person to “straighten out the question marks” and talk about his/her involuntary thoughts in order to get rid of them. Also patients’ personal resources have to be addressed through person-centered care planning in an effort to increase their sense of manageability. Because one important effect of fatigue was the feeling that *just being is enough*, it is of vital importance that the need for recuperation after MI not be neglected. Patients experiencing fatigue should be informed about the importance of rest and sleep hygiene as well as physical activity. Altogether, these efforts, along with support in dealing with problems, may help to increase patients’ motivation to manage daily life. These suggestions are in line with those of Hildingh, Fridlund, and Baigi (2008), who concluded that health-care professionals should be sensitive to patients’ sense of coherence and provide support.

Suffering an MI can be seen as an extraordinary, critical experience and may result in a psychological crisis for some persons. The crisis interrupts the person’s development, and old patterns of life are broken. Based on the old pattern, the person is driven by despair to make decisions about a new pattern (Bollnow, Katzenelson, & Myhre, 1976). The post-myocardial process described in terms of the core category *I lost the person I use to be* could be related to existential questions, and from such a perspective, the symptom fatigue may be a way to protect the person from existential thoughts. Following this line of reasoning, fatigue can be seen as a necessary adaptation to the need to be, to exist (May, 1994). However, if we are to learn more about fatigue as a possible self-protective mechanism, much more research on this existential research question is needed.

### Methodological considerations

The coding categories are generated from qualitative interviews and provide a substantiation of how the consequences of fatigue are experienced after myocardial infraction. The categories cover a large range of empirical data, and the researchers have made great efforts to gather rich data and to create strong links between data and analysis, that is, credibility. Concerning originality, the results may provide new insights, as the consequences of symptoms of fatigue after MI have not been studied previously. The criterion of resonance was reflected in the description of categories, accompanied by several citations. Regarding usefulness, these results may provide health-care professionals with new applicable knowledge in a coronary care context.

## Conclusion

This study showed that fatigue after MI had significant consequences because it restricted the informants’ potential to function in daily life as they had done previously. Managing post-MI fatigue is a personal challenge, and this study shows that something must be done to support these patients. The descriptions of fatigue that developed through the interview process may add essential information, useful to health-care professionals wishing to promote fatigue relief strategies. Support in implementing rest and sleep hygiene and physical activity is of vital importance, but not sufficient. Fatigue can be understood in light of the concepts “comprehensibility” and “manageability.” Therefore, from a person-centered perspective, health-care professionals should support patients experiencing post-MI fatigue by providing opportunities for them to straighten out the question marks and by inviting them to discuss their involuntary thoughts and feelings of being restricted in daily life functioning.
